# Schisandrin B Inhibits Cell Viability and Migration, and Induces Cell Apoptosis by circ_0009112/miR-708-5p Axis Through PI3K/AKT Pathway in Osteosarcoma

**DOI:** 10.3389/fgene.2020.588670

**Published:** 2020-12-22

**Authors:** Bing Wang, Xiaowei Wang, Xing Tong, Yingang Zhang

**Affiliations:** Department of Orthopaedics, The First Affiliated Hospital of Xi’an Jiaotong University, Xi’an, China

**Keywords:** Schisandrin B, circ_0009112, osteosarcoma, PI3K/Akt pathway, MiR-708-5p

## Abstract

Osteosarcoma is a primary tumor of bone and its incidence is increasing. Schisandrin B (Sch B), a generally used lignan in Chinese medicine, has been found to repress cancer progression. This study aims to reveal the effects and regulatory mechanism of Sch B in the viability, apoptosis and migration of osteosarcoma cells. In this study, we found circ_0009112 expression was higher and miR-708-5p expression was lower in SaOS2 and U2OS cells than in hFOB1.19 cells. Circ_0009112 expression was downregulated, but miR-708-5p was upregulated by Sch B treatment in a dose-dependent manner in SaOS2 and U2OS cells. Sch B exposure inhibited osteosarcoma development *in vitro* and *in vivo*; however, these effects were restored by circ_0009112. Furthermore, circ_0009112 acted as a sponge of miR-708-5p. Circ_0009112 regulated PI3K/AKT pathway after Sch B treatment by associating with miR-708-5p. Sch B exposure inhibited cell viability and migration, whereas promoted cell apoptosis by regulating circ_0009112/miR-708-5p axis through PI3K/AKT pathway in osteosarcoma cells. This study provided a theoretical basis for further studying osteosarcoma therapy with Sch B.

## Introduction

Osteosarcoma is an aggressive tumor of bone with tendentious metastatic ability (Makielski et al., 2019). It accounts for about 20% of bone tumors and 5% of children’s tumors (Tang et al., 2008). Osteosarcoma is more common in young people under the age of 25 with more than 55% cases, and the people aged over 40 are the second vulnerable group with 30% of cases diagnosed every year (Dorfman and Czerniak, 1995). Despite the 5 year survival rate is markedly improved after chemotherapy (Lamora et al., 2014), the mortality rate of osteosarcoma remains still high. Therefore, seeking effective medicine is necessary to osteosarcoma therapy.

Schisandrin B (Sch B) is a lignan derived from fructus schizandrae and is reported to be an efficient cure for several diseases (Nasser et al., 2020). At present, Sch B has been used to treat hepatitis, age-linked diseases and cancers (Lam and Ko, 2012; Yang et al., 2016). In 2016, Xiang et al. (2014) indicated that Sch B promoted cell apoptosis in gallbladder cancer. Revealed that Sch B participated in regulating the apoptosis in hepatoma cells Wu et al. (2004). In addition, Sch B was explained to suppress cell proliferation in lung cancer (Lv et al., 2015). Nevertheless, there were few studies on the regulatory mechanism of osteosarcoma mediated by Sch B.

Circular RNA (circRNA) is an intriguing non-coding RNA with closed loop structure and high stability (Tang and Hann, 2020). It has been unveiled that numerous circRNAs participate in the onset and development of cancer with acting as the sponge of microRNAs (miRNAs) (Hansen et al., 2013). Circ_0006332 was reported to promote tumor development by sponging miR-143 in bladder cancer (Li et al., 2019). Circ-phosphoglycerate mutase 1 (circ-PGAM1) was demonstrated to accelerate cell proliferation in ovarian cancer through binding to miR-542-3p (Zhang et al., 2020). Also disclosed that circ_0051079 promoted osteosarcoma development by modulating miR-26a-5p (Zhang et al., 2019). Exciting evidence showed circ_0009112 was one of the top 10 upregulated circRNAs in chemo-resistant osteosarcoma cell lines (Zhu et al., 2019). Thus, we guessed circ_0009112 might play a vital role in Sch-B-mediated osteosarcoma progression.

MiRNAs are a cluster of small RNAs with approximately 20 nucleotides without protein coding properties (Hua et al., 2011). And miRNAs modulate gene expression at the post-transcriptional level (Palmini et al., 2017). MiR-708-5p has been involved in cancer progression. For example, miR-708-5p repressed tumor metastasis in breast cancer (Dong et al., 2020). Declared that miR-708-5p repressed cell survival in lung cancer Wu et al. (2016). Additionally, Sui et al. (2019) revealed that miR-708-5p regulated cell proliferative and invasive abilities in osteosarcoma. In this study, we found circ_0009112 had putative binding sites for miR-708-5p.

Thus, the expression of circ_0009112 and miR-708-5p was determined in U2OS and SaOS-2 cells after Sch B treatment. The impacts of Sch B treatment on the viability, apoptosis and migration of osteosarcoma cells were revealed. In addition, whether circ_0009112 could regulate Sch B-mediated osteosarcoma progression by sponging miR-708-5p through PI3K/AKT pathway was demonstrated.

## Materials and Methods

### Cell Acquisition and Cultivation

Procell (Wuhan, China) provided human osteosarcoma cell lines (U2OS and SaOS-2) and normal osteoblast line hFOB1.19. Cells were stored in liquid nitrogen and cultured in Dulbecco’s modified Eagle’s medium (DMEM; Gibco, Carlsbad, CA, United States) with 10% fetal bovine serum (FBS; Gibco) and 1% streptomycin/penicillin (Gibco) at 37°C in an incubator with 5% CO_2_.

### Cell Transfection

The overexpression vector of circ_0009112 (circ_0009112), miR-708-5p inhibitor (anti-miR-708-5p), miR-708-5p mimic (miR-708-5p), the small interfering RNA targeting circ_0009112 (si-circ_0009112) and their controls (Vector, anti-NC, miR-NC and si-NC) were obtained from GenePharma (Shanghai, China). Sch B was purchased from Sigma (St. Louis, MO, United States). Cell transfection was performed using Lipofectamine 2000 (Thermo Fisher Scientific, Waltham, MA, United States). The sequences in this part were miR-708-5p inhibitor 5′-CCCAG CUAGAUUGUAAGCUCCUU-3′; miR-708-5p mimic 5′-AAGG AGCUUACAAUCUAGCUGGG-3′; si-circ_0009112 5′-CCAAC GATCAGGTGTTCAA-3′; anti-NC 5′-CAGUACUUUUGUGU AGUACAAA-3′; miR-NC 5′-UUUGUACUACACAAAAGUA CUG-3′ and si-NC 5′-CCATAGGACGTGTTACCAA-3′.

### Cell Counting Kit-8 Proliferation (CCK-8) Assay

SaOS2 and U2OS cells were grown in 96-well plate for 18 h. Cells were treated with Sch B, plasmids or oligonucleotides, and cultured for 24 h. Cells were incubated with 10 μl CCK-8 solution (Beyotime, Jiangsu, China) and cultured for 4 h. Cell viability was determined by assessing the absorbance of wavelength (OD = 450 nm) via a Varioskan LUX Multimode microplate reader (Thermo Fisher Scientific).

### Flow Cytometry Analysis

The apoptosis rate of SaOS2 and U2OS cells was determined via double staining kit (Solarbio, Beijing, China). Cells were harvested at 48 h after various treatments and then washed using cold phosphate buffer solution (PBS; Thermo Fisher Scientific). Cells were suspended in binding buffer. After that, 5 μl Annexin V-FITC was exposed into a centrifuge tube. After that, 5 μL propidium iodide (Pi) was added into the tube and cells were dyed for 5 min. Results were revealed by BD Accuri^TM^ C6 flow cytometry (BD Pharmingen, San Diego, CA, United States).

### Transwell Migration Assay

The migratory ability of SaOS2 and U2OS cells was detected using transwell chamber (Corning, New York, Madison, United States). SaOS2 and U2OS cells were suspended in DMEM without FBS and were added into the upper chamber. And DMEM containing 20% FBS was added into the lower chamber. Cells were cultivated for 24 h and medium was removed. Then, cells were washed with PBS, immobilized using methanol and dyed with crystal violet (Beyotime). The migrated cells were determined via counting the cell numbers on the top surface of the lower chambers by a CKX53 inverted microscope with a 100 × magnification.

### Western Blot Analysis

SaOS2 and U2OS cells were lysed using RIPA buffer (Beyotime). Protein concentration was determined and protein bands were separated by 12% sodium dodecyl sulfate PAGE. Proteins were adsorbed onto nitrocellulose membranes (Membrane Solutions, Shanghai, China). After blocked in 5% non-fat milk, proteins were incubated with anti-CyclinD1 (1:5,000, ab134175; Abcam, Cambridge, United Kingdom), anti-matrix metalloprotein 9 (anti-MMP9) (1:200, ab76003; Abcam), anti-BCL2-associated x protein (anti-Bax) (1:5,000, ab182733; Abcam), anti-phosphoinositide 3-kinase (anti-PI3K) (1:1,000, ab180967; Abcam), anti-phosphorylated PI3K (anti-p-PI3K) (1:1,000, #17366; CST, Boston, MA, United States), anti-protein kinase B (anti-AKT) (1:10,000, ab179463; Abcam), anti-phosphorylated AKT (anti-p-AKT) (1:500, ab38449; Abcam) and anti-glyceraldehyde 3-phosphate dehydrogenase (anti-GAPDH) (1:1,000, #5174; CST), respectively. After that, proteins were incubated with second antibody labeled with horse radish peroxidase (1:2,000, #7074; CST). Protein bands were visualized by RapidStep ECL Reagent (Millipore, Billerica, MA, United States). The expression of interest proteins was determined by detecting their signal intensity with an image J software (NIH, Bethesda, MD, United States). GAPDH was chosen as a reference.

### Quantitative Real Time Polymerase Chain Reaction (qRT-PCR)

SaOS2 and U2OS cells were lysed with trizol reagent (TaKaRa, Dalian, China). RNA concentration was measured by NanoDrop-1000 apparatus (Thermo Fisher Scientific). cDNA was amplified via PrimeScript RT Master Mix (Takara). For analyzing the expression of circ_0009112 and miR-708-5p, SYBR Green SuperMix (Roche, Basel, Switzerland) was employed with a Bio-Rad IQ5 thermocycler (Bio-Rad, Hercules, CA, United States). The qRT-PCR was cycled at 95°C for 20 s, 40 cycles at 95°C for 3 s, and 60°C for 20 s. The sense and antisense primers were circ_0009112 5′-ACACCTCTTCTGCTGACTGG-3′ and 5′-CT TGGAGATGGGAGCGTGTC-3′; miR-708-5p 5′-CTCACGAAG GAGCTTACAAT-3′ and 5′-ACCTCAAGAACAGTATTTCCA GG-3′; U6 5′-CTCGCTTCGGCAGCACA-3′ and 5′-AACGCTT CACGAATTTGCGT-3′; GAPDH 5′-AATGGGCAGCCGTTA GGAAA-3′ and 5′-GCGCCCAATACGACCAAATC-3′. Data were analyzed with the 2^–ΔΔCt^ method. U6 and GAPDH were chosen as controls.

### Dual-Luciferase Reporter Assay

Circbank online database was performed to assess the targeting sequence of miR-708-5p in circ_0009112. The wide-type (wt) sequence of circ_0009112 was amplified and sun-cloned into pmirGLO vector (Promega, Madison, WI, United States), and named as wt-circ_0009112. The mutant (mut) circ_0009112 without the binding sites of miR-708-5p in circ_0009112 was synthesized and inserted into pmirGLO vector (Promega), and named as mut-circ_0009112. The built plasmids were transfected into SaOS2 and U2OS cells with miR-708-5p mimic or miR-NC, respectively, with Lipofectamine 2000 (Thermo Fisher). Luciferase activities were determined by luciferase activity detection kit (Promega). *Ranilla* Luciferase activity served as a control of *firefly* luciferase activity.

### RNA Immunoprecipitation (RIP) Assay

The RIP assay was performed with a Magna RNA immunoprecipitation kit (Millipore). SaOS2 and U2OS cells were firstly lysed with RIP lysis buffer (Millipore) containing RNase inhibitor (Millipore). Lysates were then incubated with magnetic beads coated with anti-argonaute2 (anti-Ago2; ab186733; 1:50; Abcam) or anti-immunoglobulin G (anti-IgG; ab109489; 1:100; Abcam) for 24 h, respectively. After that, magnetic beads were washed and incubated with proteinase k (Millipore). Finally, the expression levels of circ_0009112 and miR-708-5p were determined by qRT-PCR.

### RNA Pull-Down Assay

Biotinylated miR-708-5p (Bio-miR-708-5p) and miR-NC (Bio-miR-NC) were provided by Sangon (Shanghai, China), and were transfected into SaOS2 and U2OS cells for 48 h. Cultured cells were collected and lysed with RIP lysis buffer (Millipore). Then, streptavidin-coupled beads (Invitrogen, Carlsbad, CA, United States) were used to incubate with lysates. Finally, relative circ_0009112 enrichment by detected by qRT-PCR.

### *In vivo* Assay

Charles River (Beijing, China) provided the 5 week old male BALB/c nude mice. All mice were fed in pathogen-free environment, and were randomly divided into 3 groups (*N* = 6, respectively). 5 × 10^6^ SaOS2 cells stably transfected with Vector or circ_0009112 were injected into the tail vein of mice. At 48 h after injection, mice were intraperitoneally administrated with Sch B (Sigma; 40 mg/kg) every 2 days until the end of the experiment with PBS (Thermo Fisher Scientific) as a control. At the seventh day, tumor volume was measured every 7 days. At the 28th day, all mice were killed. Forming tumors were excised, and tumor weight was determined. Additionally, a part of every tumor was kept for further illustrating the impacts of circ_0009112 on the expression of circ_0009112 and miR-708-5p. The Animal Care and Use Committee of The First Affiliated Hospital of Xi’an Jiaotong University agreed with this study.

### Data Analysis

Data were analyzed with SPSS 21.0 software (IBM, Somers, NY, United States). Every experiment was conducted at least three times. Data were presented as means + standard deviations. Significant differences were compared by two-tailed Student’s *t*-tests between two groups or one-way analysis of variance among three or more groups. Statistical significance was defined when *P* < 0.05.

## Results

### Sch B Treatment Repressed Cell Viability and Migration, Whereas Induced Cell Apoptosis in SaOS2 and U2OS Cells

**FIGURE 1:**
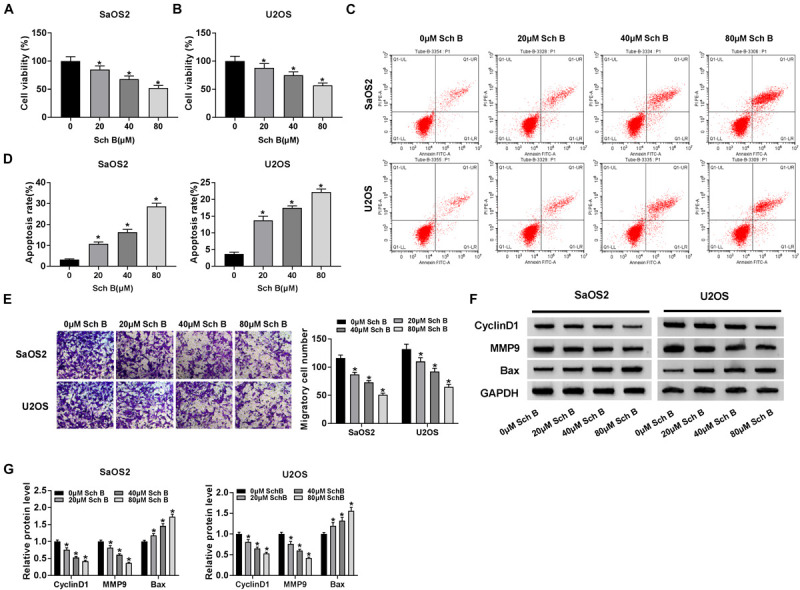
Schisandrin B treatment repressed osteosarcoma development. **(A,B)** The effect of Sch B (20, 40, and 80 μM) on cell viability was revealed by CCK-8 assay in SaOS2 and U2OS cells. **(C,D)** The impact of Sch B (20, 40, and 80 μM) on the apoptosis of SaOS2 and U2OS cells was unveiled by flow cytometry analysis. **(E)** Transwell migration assay was performed to determine the effect of Sch B (20, 40, and 80 μM) on cell migration. **(F,G)** Western blot was carried out to detect the effects of Sch B (20, 40, and 80 μM) on the protein expression of CyclinD1, MMP9 and Bax in SaOS2 and U2OS cells. *P < 0.05.

the effects of Sch B (20, 40, and 80 μM) on cell viability, apoptosis and migration were firstly explored in SaOS2 and U2OS cells. CCK-8 assay demonstrated that Sch B treatment repressed cell viability in a dose-dependent manner in SaOS2 and U2OS cells (Figures 1A,B) (The *P*-values in figures were provided in Supplementary Table 1). Flow cytometry analysis revealed that Sch B exposure induced cell apoptosis in a concentration-dependent manner in SaOS2 and U2OS cells (Figures 1C,D). Transwell migration assay showed that cell migratory ability was suppressed after Sch B treatment in a dose-dependent manner (Figure 1E). Meanwhile, the effects of Sch B (20, 40, and 80 μM) on the protein levels of CyclinD1, MMP9 and Bax were disclosed by western blot. Results unveiled that Sch B exposure repressed the protein levels of CyclinD1 and MMP9, while upregulated Bax protein expression in a concentration-dependent manner in SaOS2 and U2OS cells (Figures 1F,G). These data showed that Sch B played a negatively regulatory role in osteosarcoma progression.

### Circ_0009112 Expression Was Downregulated and miR-708-5p Expression Was Upregulated After Sch B Treatment in SaOS2 and U2OS Cells

**FIGURE 2:**
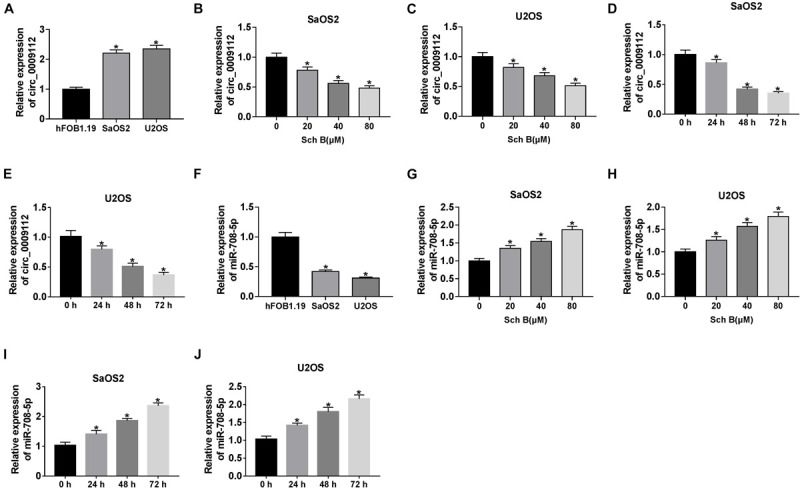
Schisandrin B downregulated circ_0009112 and upregulated miR-708-5p expression in SaOS2 and U2OS cells. **(A,F)** Circ_0009112 and miR-708-5p expression were determined by qRT-PCR in hFOB1.19, SaOS2 and U2OS cells. **(B,C)** The effect of Sch B (20, 40, and 80 μM) on circ_0009112 expression was determined by qRT-PCR in SaOS2 and U2OS cells. **(D,E)** The impact of Sch B (80 μM) on circ_0009112 expression was revealed by qRT-PCR at 24, 48, and 72 h after transfection. **(G,H)** The impact of Sch B treatment (20, 40, and 80 μM) on miR-708-5p expression was investigated by qRT-PCR in SaOS2 and U2OS cells. **(I,J)** The impact of Sch B (80 μM) on miR-708-5p expression was determined by qRT-PCR at 24, 48, and 72 h after transfection. *P < 0.05.

Circ_0009112 expression was firstly determined in SaOS2 and U2OS cells. QRT-PCR results showed that circ_0009112 expression was upregulated in SaOS2 and U2OS cells compared with hFOB1.19 cells (Figure 2A). The impact of Sch B exposure on circ_0009112 expression was further determined in SaOS2 and U2OS cells. QRT-PCR results showed that circ_0009112 expression was downregulated by Sch B exposure in a dose-dependent manner in SaOS2 and U2OS cells (Figures 2B,C). Additionally, Sch B treatment (80 μM) downregulated circ_0009112 expression at 24, 48, and 72 h after transfection as compared to control groups in SaOS2 and U2OS cells (Figures 2D,E). Meanwhile, qRT-PCR revealed that miR-708-5p expression was lower in SaOS2 and U2OS cells than that in hFOB1.19 cells (Figure 2F). And miR-708-5p expression was upregulated by Sch B in a concentration-dependent manner in SaOS2 and U2OS cells (Figures 2G,H). In addition, miR-708-5p expression was upregulated by Sch B exposure (80 μM) after 24 h since transfection when compared with control groups in SaOS2 and U2OS cells (Figures 2I,J). These results suggested that the effects of Sch B on osteosarcoma progression might be regulated by circ_0009112 and miR-708-5p.

### Circ_0009112 Reversed the Effects of Sch B on the Growth of Osteosarcoma *in vitro* and *in vivo*

**FIGURE 3:**
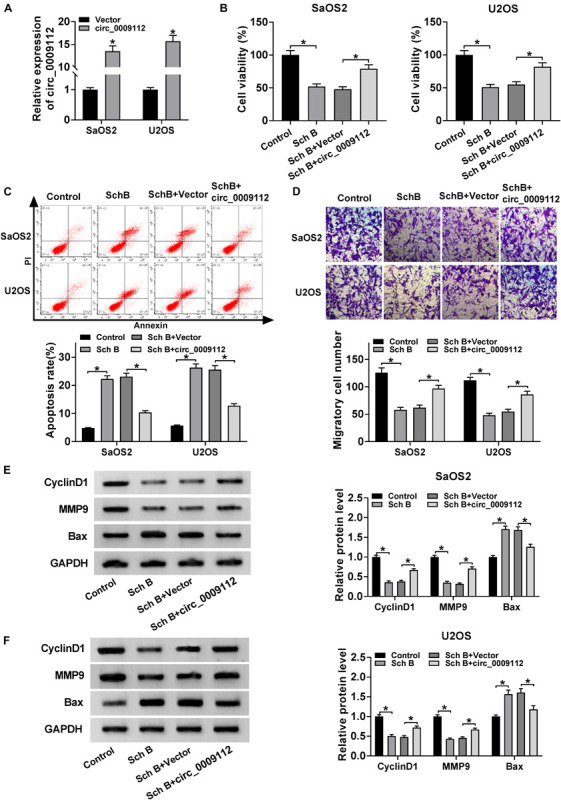
circ_0009112 overexpression attenuated the inhibition effects of Sch B on osteosarcoma progression. **(A)** The transfection efficiency of circ_0009112 was revealed by qRT-PCR in SaOS2 and U2OS cells. **(B)** The effect of circ_0009112 overexpression on the viability of SaOS2 and U2OS cells after Sch B treatment was determined by CCK-8 assay. **(C)** Flow cytometry assay was performed to detect the impact of circ_0009112 on cell apoptosis after Sch B exposure in SaOS2 and U2OS cells. **(D)** Transwell migration assay was performed to reveal the effects between circ_0009112 and Sch B treatment on the migration of SaOS2 and U2OS cells. **(E,F)** Western blot was used to elucidate the effects between circ_0009112 and Sch B exposure on the protein expression of CyclinD1, MMP9 and Bax in SaOS2 and U2OS cells. *P < 0.05.

To further explore the effects of circ_0009112 on Sch B-mediated osteosarcoma development, the transfection efficiency of circ_0009112 was determined in SaOS2 and U2OS cells. QRT-PCR result revealed that circ_0009112 expression was dramatically upregulated after circ_0009112 transfection in SaOS2 and U2OS cells (Figure 3A). Subsequently, the effects of circ_0009112 overexpression on cell viability, apoptosis and migration after Sch B exposure were disclosed. CCK-8 assay showed that Sch B treatment repressed cell viability in SaOS2 and U2OS cells, whereas circ_0009112 overexpression restored this effect (Figure 3B). Flow cytometry analysis evaluated that Sch B exposure induced cell apoptosis; however, this effect was reversed by circ_0009112 overexpression in SaOS2 and U2OS cells (Figure 3C). Transwell migration assay also elucidated that circ_0009112 overexpression hindered the inhibition effect of Sch B on the migration of SaOS2 and U2OS cells (Figure 3D). In addition, western blot analysis revealed that ectopic circ_0009112 expression restrained the inhibition effects of Sch B treatment on CyclinD1 and MMP9 protein expression, and promotion effect of that on Bax protein expression in SaOS2 and U2OS cells (Figures 3E,F). Meanwhile, the effects between Sch B treatment and circ_0009112 on tumor formation *in vivo* were revealed. Results showed circ_0009112 overexpression hindered the inhibitive impacts of Sch B on tumor volume and weight (Supplementary Figures S2A,B). Furthermore, qRT-PCR data presented the repressive impact of Sch B on circ_0009112 expression and the promotive influence of that on miR-708-5p expression were also restored after circ_0009112 overexpression *in vivo* (Supplementary Figures S2C,D). Overall, all evidences showed that the effects of Sch B on osteosarcoma process could be regulated by circ_0009112.

### MiR-708-5p Inhibitor Relieved the Effects of Sch B on Cell Viability, Apoptosis and Migration in SaOS2 and U2OS Cells

**FIGURE 4:**
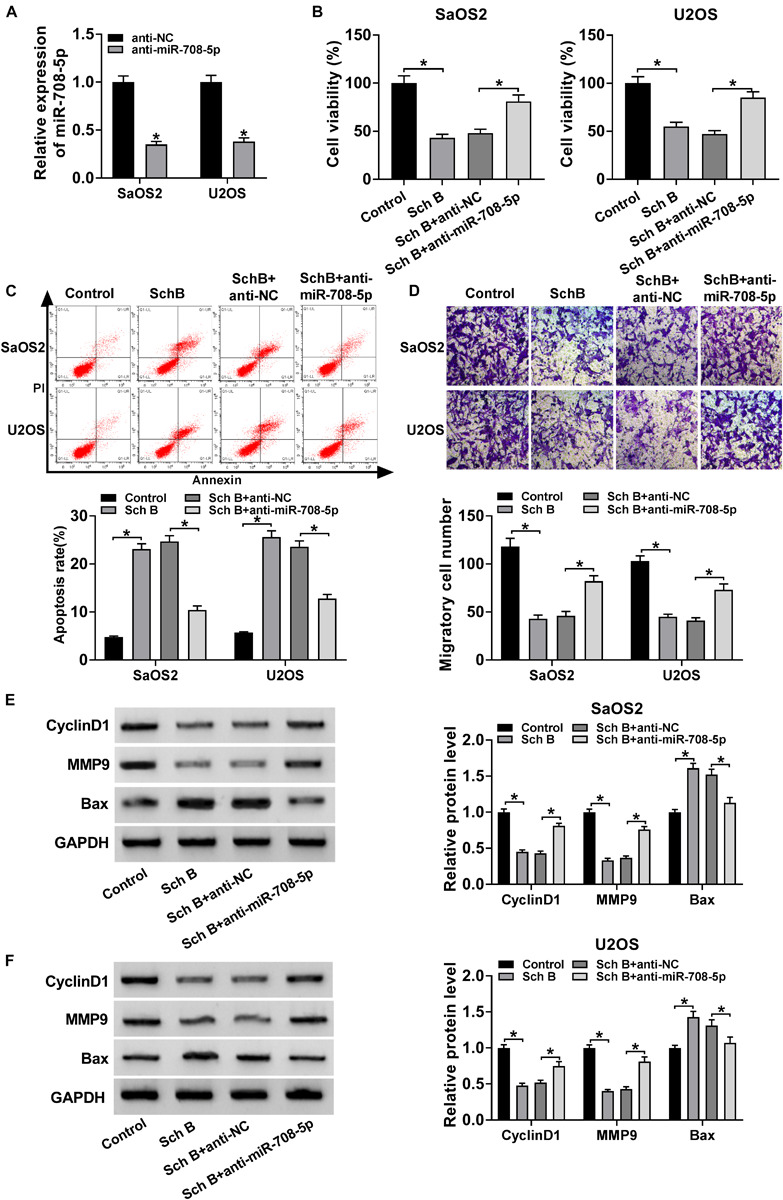
MiR-708-5p inhibitor restored the effects of Sch B on cell viability, apoptosis and migration. **(A)** QRT-PCR was performed to determine the interfering efficiency of anti-miR-708-5p in SaOS2 and U2OS cells. **(B)** CCK-8 assay was employed to demonstrate the effects between miR-708-5p inhibitor and Sch B exposure on the viability of SaOS2 and U2OS cells. **(C)** Flow cytometry analysis was carried out to explain the impacts between miR-708-5p inhibitor and Sch B exposure on cell apoptosis. **(D)** Transwell migration assay was used to elucidate the effect of anti-miR-708-5p on cell migration after Sch B treatment in SaOS2 and U2OS cells. **(E,F)** Western blot was performed to unveil the effects between miR-708-5p inhibitor and Sch B treatment on the protein expression of CyclinD1, MMP9 and Bax in SaOS2 and U2OS cells. *P < 0.05.

The effects of miR-708-5p on Sch B-mediated cell viability, apoptosis and migration were determined in this part. QRT-PCR was conducted to detect the interfering efficiency of miR-708-5p inhibitor, and results showed that miR-708-5p expression was downregulated by miR-708-5p inhibitor in SaOS2 and U2OS cells (Figure 4A). Subsequently, CCK-8 assay showed that Sch B treatment repressed cell viability in SaOS2 and U2OS cells, but this effect was reversed by miR-708-5p inhibitor (Figure 4B). Flow cytometry assay revealed that the apoptotic rate of SaOS2 and U2OS cells was upregulated by Sch B treatment, whereas this impact was impeded by miR-708-5p inhibitor (Figure 4C). Transwell migration assay revealed that Sch B exposure suppressed cell migration; however, this inhibition effect was restrained by anti-miR-708-5p (Figure 4D). Additionally, western blot assay demonstrated that miR-708-5p inhibitor attenuated the inhibition effect of Sch B on the protein expression of CyclinD1 and MMP9, and the promotion effect of Sch B exposure on Bax expression in SaOS2 and U2OS cells (Figures 4E,F). These data demonstrated that miR-708-5p participated in the regulation of Sch B-mediated cell viability, apoptosis and migration in SaOS2 and U2OS cells.

### Circ_0009112 Acted as a Sponge of miR-708-5p

**FIGURE 5:**
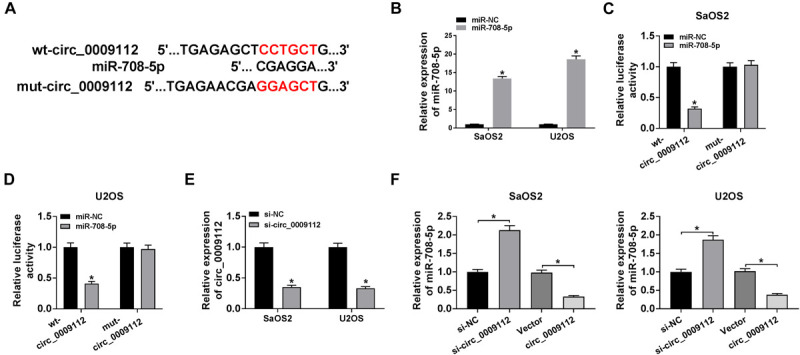
Circ_0009112 bound to miR-708-5p in SaOS2 and U2OS cells. **(A)** The binding sequence of miR-708-5p in circ_0009112 was predicted by circbank online database. **(B)** The effect of miR-708-5p mimic on miR-708-5p expression was determined by qRT-PCR. **(C,D)** Luciferase activity was detected by dual-luciferase reporter assay in SaOS2 and U2OS cells. **(E)** QRT-PCR was performed to reveal the effect of si-circ_0009112 on circ_0009112 expression. **(F)** The impacts of circ_0009112 knockdown or overexpression on miR-708-5p expression were unveiled by qRT-PCR in SaOS2 and U2OS cells. *P < 0.05.

Given the expression of circ_0009112 and miR-708-5p in SaOS2 and U2OS cells after Sch B treatment, and the effects of circ_0009112 and miR-708-5p on Sch B-mediated osteosarcoma progression, the relationship between circ_0009112 and miR-708-5p was revealed. Circbank online database showed that circ_0009112 contained the binding sites of miR-708-5p (Figure 5A). QRT-PCR result showed that miR-708-5p expression was dramatically upregulated after miR-708-5p mimic transfection (Figure 5B). Subsequently, dual-luciferase reporter assay revealed that luciferase activity was significantly repressed after wt-circ_0009112 and miR-708-5p co-transfection in SaOS2 and U2OS cells, whereas that had no obvious change in mut-circ_0009112 + miR-708-5p group (Figures 5C,D). Meanwhile, RIP assay displayed circ_0009112 and miR-708-5p were dramatically enriched by anti-Ago2 as compared to anti-IgG (Supplementary Figure S1A). RNA pull-down assay showed circ_0009112 was obviously enriched by Bio-miR-708-5p when compared with control group (Supplementary Figure S1B). In addition, the effects of circ_0009112 knockdown and overexpression on miR-708-5p expression were investigated by qRT-PCR. The knockdown efficiency of si-circ_0009112 was firstly detected by qRT-PCR, and result showed that circ_0009112 expression was downregulated by si-circ_0009112 (Figure 5E). Then, results demonstrated that circ_0009112 knockdown obviously upregulated miR-708-5p expression and that circ_0009112 overexpression apparently downregulated miR-708-5p expression in SaOS2 and U2OS cells (Figure 5F). These data showed that circ_0009112 was associated with miR-708-5p in SaOS2 and U2OS cells.

### Circ_0009112 Abolished Sch B-Mediated Effects on Cell Viability, Apoptosis, and Migration by Sponging miR-708-5p

**FIGURE 6:**
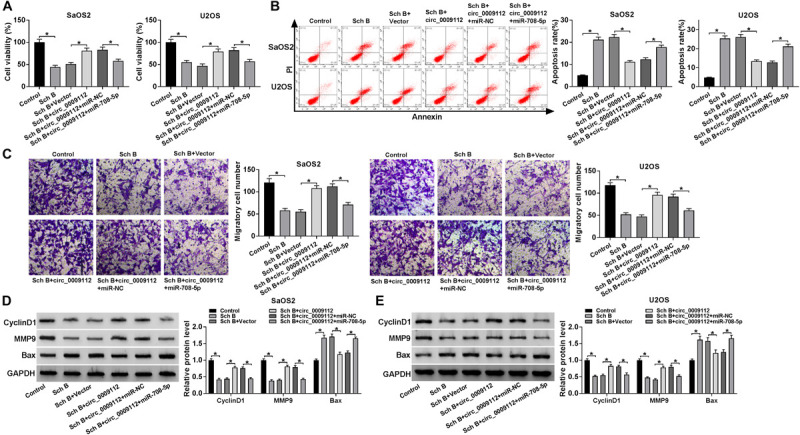
Circ_0009112 promoted cell viability and migration, while repressed cell apoptosis after Sch B exposure by binding to miR-708-5p. **(A)** The effects between circ_0009112 and miR-708-5p on cell viability after Sch B treatment were shown by CCK-8 assay in SaOS2 and U2OS cells. **(B)** Flow cytometry was performed to evaluate the impacts between circ_0009112 and miR-708-5p on cell apoptosis after Sch B exposure in SaOS2 and U2OS cells. **(C)** Transwell migration assay was employed to explain the impacts between circ_0009112 and miR-708-5p on Sch B-mediated inhibition effect on cell migration in SaOS2 and U2OS cells. **(D,E)** The effects between circ_0009112 and miR-708-5p on protein expression of CyclinD1, MMP9 and Bax after Sch B exposure were unveiled by western blot. *P < 0.05.

Circ_0009112 has been revealed to bind to miR-708-5p, the effects between circ_0009112 and miR-708-5p on cell viability, apoptosis and migration after Sch B treatment were further studied. CCK-8 assay presented that circ_0009112 overexpression hindered Sch B-mediated inhibition effect on cell viability in SaOS2 and U2OS cells, whereas this effect was restored by miR-708-5p mimic (Figure 6A). Flow cytometry assay unveiled that circ_0009112 overexpression restrained Sch B-mediated promotion effect on cell apoptosis in SaOS2 and U2OS cells, while this impact was relieved by miR-708-5p (Figure 6B). Transwell migration assay elucidated that ectopic circ_0009112 expression attenuated the inhibition effect of Sch B treatment on cell migration; however, this effect was impaired by miR-708-5p (Figure 6C). Meanwhile, western blot results revealed that circ_0009112 hindered the inhibition effects of Sch B exposure on CyclinD1 and MMP9 protein expression, and the promotion impact of Sch B treatment on Bax protein expression; but these effects were abolished by miR-708-5p in SaOS2 and U2OS cells (Figures 6D,E). These data revealed that circ_0009112 regulated Sch B-mediated impacts on cell viability, apoptosis and migration by associating with miR-708-5p in osteosarcoma.

### Circ_0009112 Regulated PI3K/AKT Pathway After Sch B Treatment by Sponging miR-708-5p

**FIGURE 7:**
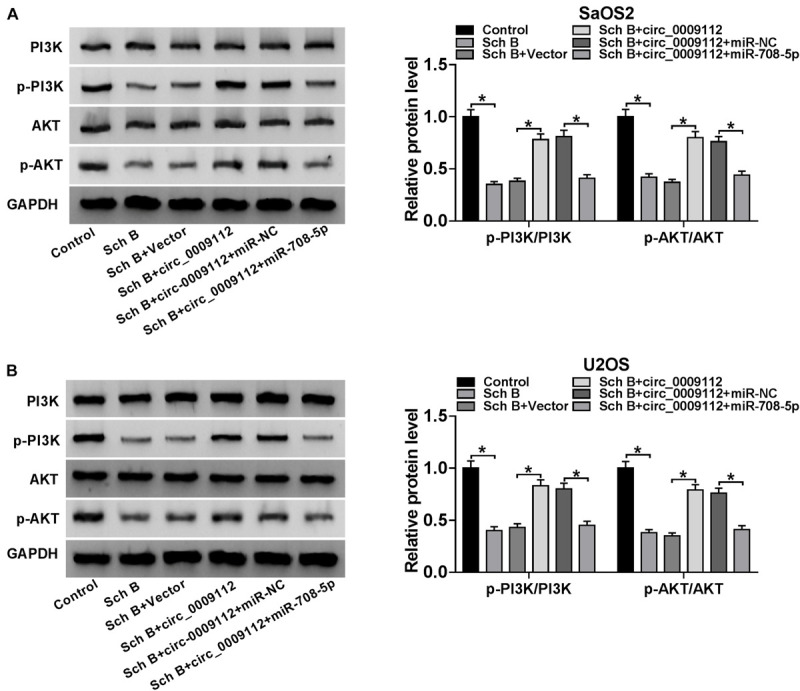
Circ_0009112 regulated Sch B-mediated osteosarcoma development by associating with miR-708-5p through PI3K/AKT signaling pathway. **(A,B)** Western blot was employed to explain the effects among Sch B, circ_0009112 and miR-708-5p on protein expression of PI3K, p-PI3K, AKT, and p-AKT in SaOS2 and U2OS cells. *P < 0.05.

In order to determine whether Sch B-mediated osteosarcoma progression regulated by circ_0009112 and miR-708-5p was related to PI3K/AKT pathway, the effects among Sch B, circ_0009112 and miR-708-5p on protein expression of PI3K, p-PI3K, AKT and p-AKT were revealed by western blot. Results showed that Sch B-mediated inhibition effects on the protein expression of p-PI3K and p-AKT were restrained by circ_0009112 in SaOS2 and U2OS cells, whereas these impacts were attenuated by miR-708-5p (Figures 7A,B). These results demonstrated that circ_0009112 modulated Sch B-mediated osteosarcoma development by binding to miR-708-5p through PI3K/AKT signaling pathway.

## Discussion

Osteosarcoma is a main malignant bone cancer and poses a heavy burden to the health of children and adolescents (Sampson et al., 2015). Sch B, extracted from *Schisandra chinensis*, is found to inhibit cancer progression (Xiang et al., 2014). Cao et al. (2019) indicated Sch B attenuated renal fibrosis by regulating miR-30e. Mou et al. (2019) also explained Sch B suppressed the development of diabetic nephropathy by modulating inflammatory response and oxidative stress. However, its regulatory mechanism in osteosarcoma evolution is still unclear. Herein, the influences of Sch B on cell viability, apoptotic rate and migratory ability in osteosarcoma and underneath mechanism were revealed.

Sch B was unveiled to suppress cell proliferation and induce cell cycle arrest in gastric cancer (Liu et al., 2007). Yang et al. (2016) revealed that Sch B restrained cell viability and accelerated cell apoptosis in cholangiocarcinoma. In our experiments, the influences of Sch B on cell viability, apoptosis and migratory ability were revealed in osteosarcoma. From our findings, Sch B was exhibited to repress cell viability and induce cell apoptosis in osteosarcoma. Beyond that, Sch B was shown to repress cell migration and downregulate circ_0009112 expression in osteosarcoma. Then, the effects of circ_0009112 on osteosarcoma progression were explored. Data presented that its overexpression accelerated cell viability and migration, whereas repressed cell apoptosis in osteosarcoma. Meanwhile, our findings displayed circ_0009112 overexpression reversed the repressive impacts of Sch B treatment on osteosarcoma development *in vitro* and *in vivo*. These results demonstrated circ_0009112 acted as an oncogene in osteosarcoma evolution, and circ_0009112 could modulate Sch B-mediated impacts on osteosarcoma process.

Current research disclosed circRNAs commonly served as competing endogenous RNAs to regulate miRNA expression to further mediate cancer processes (Huang et al., 2016). In this study, circ_0009112 was revealed to act as a sponge of miR-708-5p. Previous study displayed miR-708-5p overexpression repressed cell viability and migration, and promoted cell apoptosis in osteosarcoma (Sui et al., 2019). Feng et al. (2020) also investigated that miR-708-5p hindered cell migration and invasion in osteosarcoma. Similarly, in our view, miR-708-5p inhibitor attenuated the inhibition effects of Sch B on cell viability and migration, and the promotion effect of Sch B on cell apoptosis, which suggested that miR-708-5p hindered cell viability and migration, but induced cell apoptosis in osteosarcoma. In addition, miR-708-5p expression was revealed to be upregulated after Sch B treatment in osteosarcoma cells.

The regulatory mechanism of Sch B on cancer progression is enrolled in signaling pathway. For instance, Ran et al. evaluated that Sch B alleviated osteoarthritis by repressing NF-κB and MAPK pathways (Ran et al., 2018). Liu et al. (2018) explained that dichlorodiammine platinum (DDP) promoted cell apoptosis, whereas Sch B repressed this effect by regulating ERK/NF-κB pathway. Our data showed that Sch B exposure downregulated the expression of PI3K/AKT pathway-related proteins (p-PI3K and p-AKT), which meant that Sch B regulated osteosarcoma progression by blocking PI3K/AKT signaling pathway. In addition, we also explained that Sch B-mediated inhibition effects on PI3K/AKT pathway were attenuated by circ_0009112, whereas these impacts were restored by miR-708-5p.

Collectively, Sch B treatment downregulated circ_0009112 and upregulated miR-708-5p expression in SaOS2 and U2OS cells. Sch B exposure suppressed cell viability and migration, whereas accelerated cell apoptosis in osteosarcoma. Additionally, both circ_0009112 overexpression and miR-708-5p inhibitor restrained the impacts of Sch B on osteosarcoma progression. Furthermore, Sch B treatment inactivated PI3K/AKT signaling pathway through regulating circ_0009112/miR-708-5p axis. These results provide a theoretical basis for circ_0009112 application as a promising therapeutic target and predictive marker of osteosarcoma. Furthermore, these evidences also indicate circ_0009112 can be considered in further developing Sch B-mediated therapy of osteosarcoma.

## Data Availability Statement

The original contributions presented in the study are included in the article/Supplementary Material, further inquiries can be directed to the corresponding author/s.

## Ethics Statement

The animal study was reviewed and approved by The First Affiliated Hospital of Xi’an Jiaotong University.

## Author Contributions

XW designed the study. BW conducted the experiments. XT collected and analyzed the data. YZ drafted the manuscript. All authors contributed to the article and approved the submitted version.

## Conflict of Interest

The authors declare that the research was conducted in the absence of any commercial or financial relationships that could be construed as a potential conflict of interest.

## Supplementary Material

Supplementary Figure 1 Circ_0009112 was directly associated with miR-708-5p. **(A,B)** RIP and RNA pull-down assays were conducted to demonstrate circ_0009112 directly interacted with miR-708-5p in SaOS2 and U2OS cells.

Supplementary Figure 2 Circ_0009112 overexpression restored the impact of Sch B treatment on tumor formation *in vivo*. **(A,B)** The effects between Sch B treatment and circ_0009112 overexpression on the volume and weight of tumors were revealed. **(C,D)** QRT-PCR analysis was employed to determine the influences between Sch B treatment and circ_0009112 overexpression on the expression of circ_0009112 and miR-708-5p.

Supplementary Table 1 The *P*-values in figures.
