# Supplementary figures and images for: Schisandrin B Inhibits Cell Viability and Migration, and Induces Cell Apoptosis by circ_0009112/miR-708-5p Axis Through PI3K/AKT Pathway in Osteosarcoma
Source: Front Genet. 2020 Dec 22;11:588670. doi: 10.3389/fgene.2020.588670 (PMC7783358; doi:10.3389/fgene.2020.588670)

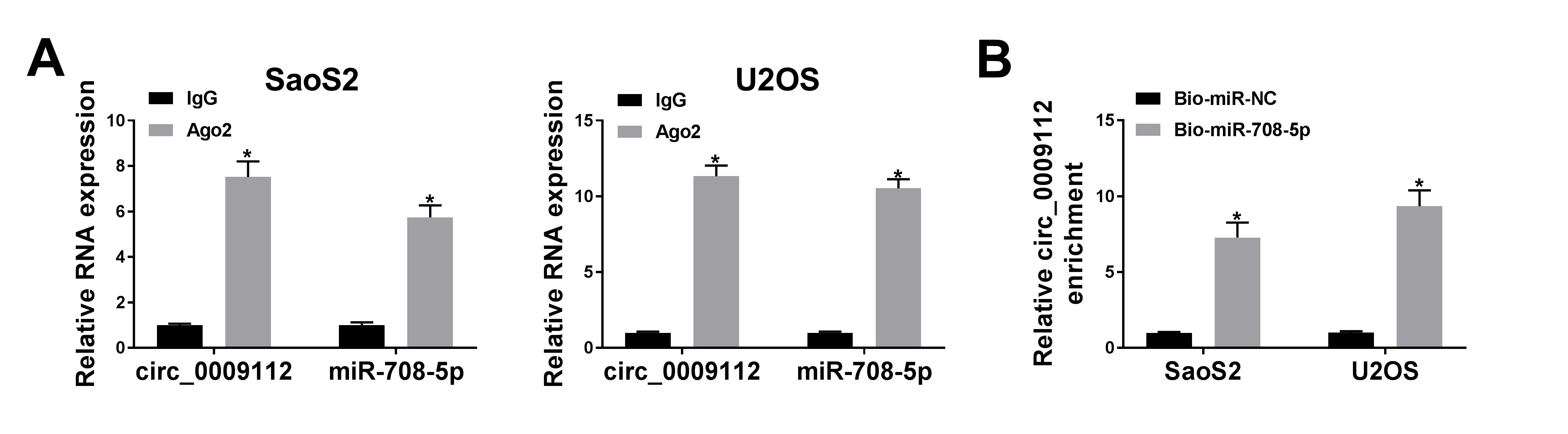

Supplement: Supplementary Figure 1 — Circ_0009112 was directly associated with miR-708-5p. (A,B) RIP and RNA pull-down assays were conducted to demonstrate circ_0009112 directly interacted with miR-708-5p in SaOS2 and U2OS cells. [file Image_1.TIF]

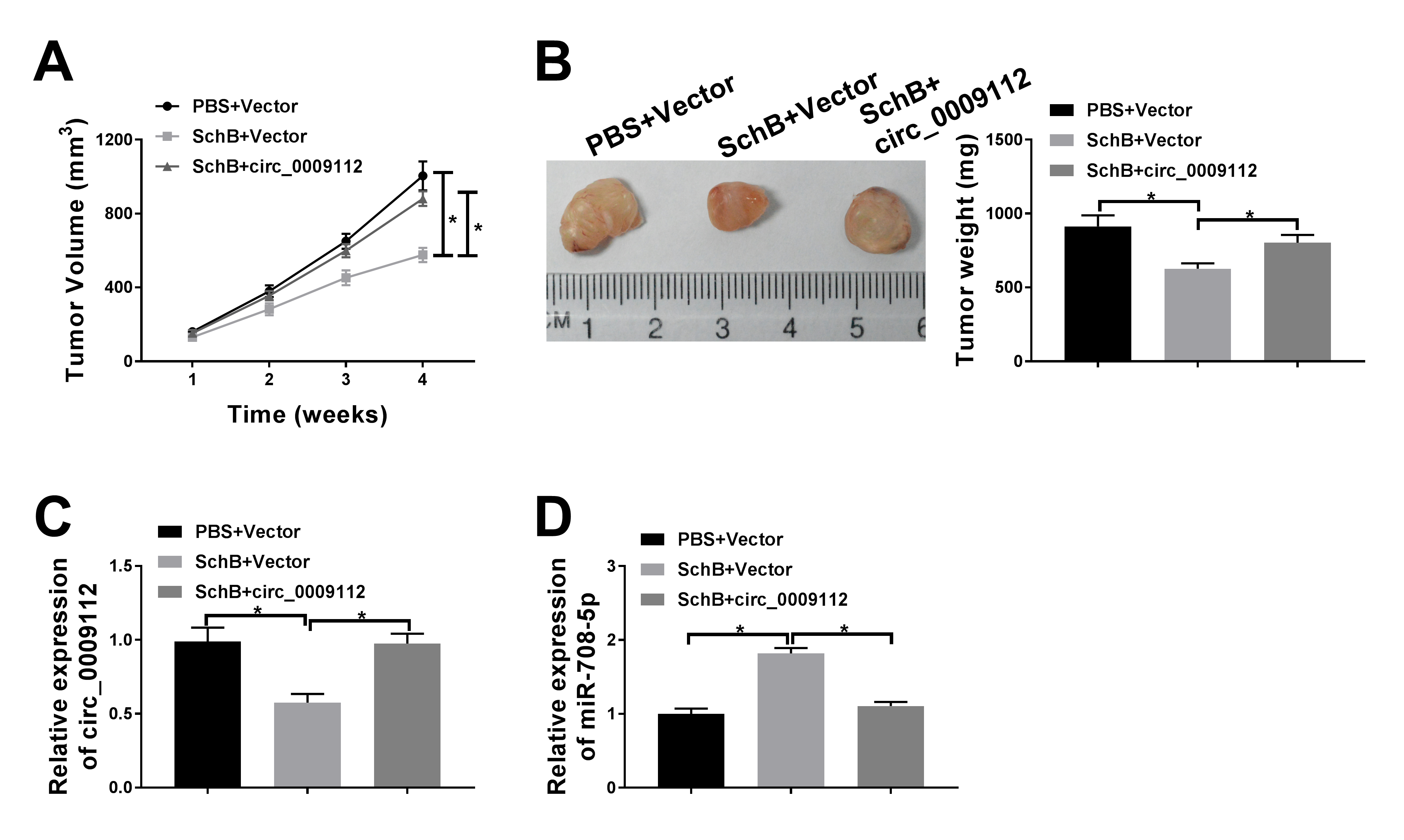

Supplement: Supplementary Figure 2 — Circ_0009112 overexpression restored the impact of Sch B treatment on tumor formation in vivo. (A,B) The effects between Sch B treatment and circ_0009112 overexpression on the volume and weight of tumors were revealed. (C,D) QRT-PCR analysis was employed to determine the influences between Sch B treatment and circ_0009112 overexpression on the expression of circ_0009112 and miR-708-5p. [file Image_2.TIF]
